# Economy in Coal

**Published:** 1901-01-12

**Authors:** G. E. M. Vaughan


### EDITOR'S LETTER-BOX.

[Our Correspondents are reminded that prolixity is a great bar to publication, and that brevity of style and conciseness of statement greatly facilitate early insertion.]

### ECONOMY IN COAL.

To the Editor of *The Hospital*.

DEAR SIR,—I should be very glad if you could tell me (1) where the patent "paraffin coke" mentioned by your correspondent on "How to Lessen Coal Bills" (December 15th) can be obtained; and (2) how the difficulty is overcome, when using gas jets for a coke or coal fire, in an ordinary fire basket, of dust falling into the burners? In my stove, just fitted with the newest kind of burner, the opening of each burner is as large as a threepenny bit, and I do not see how they are to be prevented from getting clogged with dust or small particles. I shall be very grateful if your correspondent can tell me, as I do not like the asbestos fire, and should like to substitute coal or coke, keeping the burners for the sake of cleanliness and saving of labour.

I am, yours faithfully,

G. E. M. VAUGHAN.

This question the writer of the article referred to answers:—

I obtain my "paraffin coke" from the "Iron ers'Found Grinding Company", 3 West Scotland Street, Glasgow. I do not know if the company has any depot in London, but they would doubtless give your correspondent all information on the subject. As the coke burns away practically entirely, the dust trouble has caused me very little inconvenience. Occasionally, when a burner seemed to be throwing out less gas than usual, it has been cleaned out with a hair-pin—not a very serious operation. But it must be pointed out that I do not use "atmospheric" burners. When gas burns against coke, which is itself burning, there seems to be no necessity for any admixture of air. I use the pure gas just as it issues from a series of holes bored in the side of the pipe. I use no "burners" at all, simply perforations in the pipe. The necessary preparations for arranging an ordinary grate to burn this fuel are very simple. Have an iron plate made to fit—but rather loosely than the reverse—above the bars at the bottom of the grate, and lead the gas through a pipe with some half-dozen perforations along the interior of the front bars. I would accentuate the desirability of simplicity, for I find that plumbers prefer to insert more elaborate arrangements, which, in my experience, prevent the coke igniting readily, and make any cleaning of the grate itself or of the tiles of the hearth very difficult. The first coke fire I had put in is an example of that. The plumber removed the bottom bars entirely, which was quite unnecessary; then placed an immovable iron plate considerably above the gas-jets, this plate being cut in niches to admit the flame. He also placed a row of tubes at the back of the fire-place, the usefulness of which I have never yet been able to find out. But this fire has never burnt so well as the next which I had put in, in which the arrangement is the very simple and, I should add, inexpensive one I spoke of first. I mention this as one or two friends who have installed these fires have found a difficulty in making their plumbers understand that the thing can be done quite simply, and without much cost. For those who have not and do not desire a permanent gas pipe in their fireplace, there is an arrangement of rubber tubing with a fork at the end, a burner being at each end of the prongs of the fork. This can be connected at will with a gas pipe in the room, which is usually hidden by a brass plate, and taken away when the coke is ignited. Of course this is not so convenient for livening up the fire again if it has got low or gone out, but if the fire is replenished in time no gas should be required after the first lighting. I ought to mention that the coke, following the price of coal, has become much dearer lately. The last I got in cost me £3 per ton, just twice what it cost two years ago. But as there is absolutely no waste, it is not really an extravagant fuel. It gives out a very intense heat, for which reason I prefer to have the permanent row of gas-jets in the grate, as sometimes my room would get too hot if the fire were constantly replenished with the paraffin coke. I prefer to let it get quite low and relight the coke with the gas. I will gladly answer any other queries regarding this fire which your correspondent may have to put.

